# Research progress of colorectal cancer in genomic and transcriptomic at multi-level

**DOI:** 10.3389/fgene.2025.1533817

**Published:** 2025-05-30

**Authors:** Qinglian Wen, Shuangyan Han, Yongxia Cui

**Affiliations:** ^1^ Department of Radiation Oncology, Cancer Center, West China Hospital, Sichuan University, Chengdu, Sichuan, China; ^2^ Department of Oncology, The Affiliated Hospital of Southwest Medical University, Luzhou, Sichuan, China

**Keywords:** evolutionary genomics, single-cell genomics, tumor heterogeneity, tumor microenvironment, CRC (colorectal cancer)

## Abstract

Colorectal cancer is a common malignant tumor in the gastrointestinal tract, and the mechanisms of its occurrence, development, and metastasis have always been the focus of the medical community’s attention. The study of CRC genetic mechanisms began with the identification of oncogenes or tumor suppressor genes and their key pathways. With further research, researchers gradually realized that single genes or pathways alone could not explain the occurrence, development, and metastasis of CRC. The development of bulk sequencing technology has helped us to analyze the occurrence, development, and metastasis mechanisms of CRC from a multi-gene, multi-pathway, and multi-dimensional perspective, but it has not brought significant benefits to the clinical treatment of tumors. The main reason for this is that bulk sequencing technology relies on homogeneous cell grouping and cannot capture the heterogeneity between cells within the tumor and the interactions within the tumor microenvironment. The development of single-cell technology has made it possible to study the mechanisms of heterogeneity between cells within CRC and the interaction within the tumor microenvironment. This review discusses the mechanisms of CRC occurrence and development in three stages: traditional molecular biology level of single gene, bulk sequencing, and single-cell sequencing. These results show that the occurrence of CRC is the result of complex interactions between genetic and non-genetic factors in somatic cell evolution, where the heterogeneity between cells within the tumor and the tumor microenvironment are crucial for CRC progression.

## 1 Introduction

Colorectal cancer (CRC) is a common malignant tumor in the gastrointestinal tract, with an incidence and mortality rate second only to gastric cancer, esophageal cancer, and primary liver cancer in malignant tumors of the digestive system. As a complex and serious disease, CRC has always been the focus of attention in the medical field for its occurrence and metastasis mechanisms. With the rapid development of multi-omics technologies and bioinformatics, it has become possible to analyze the pathogenesis of CRC in higher dimensions, and human understanding of CRC has reached an unprecedented extension (William et al., 2011).

### 1.1 Molecular biology research at the single gene level in colorectal cancer

The occurrence and development of CRC have undergone a series of molecular changes, among which chromosomal instability (CIN), Microsatellite instability (MSI), and DNA hypermethylation CpG Island methylator phenotype, (CIMP) are considered to be the main mechanisms leading to the occurrence and development of CRC (Malki et al., 2020). Among them, early-onset colorectal cancer (EOCRC) presents higher levels of MSI in most anatomical sites, but the proportion of CIMP-high and BRAF mutations is relatively low (Ugai et al., 2023), and EOCRC tumors often have lower levels of tumor-infiltrating lymphocytes, and peritumoral lymphocytic reaction (Ugai et al., 2022). Additionally, the mutation of the tumor suppressor gene *APC* will activate the Wnt signaling pathway, which is believed to be the initial event of CRC, in which *APC’s* 1039th codon 5bp base deletion exists in somatic cell mosaic phenomenon, speculated as one of the reasons for the early onset of CRC (onset age <50 years) (Gong, 2015). Additionally, during the development of CRC, there is also involvement of tumor suppressor gene deletions such as *TP53, KRAS, PIK3CA, PCDH3B*, and *PTEN* (Clarens, 2004), as well as the continuous activation of oncogenes, leading to the dysregulation of multiple signaling pathways such as Wnt, MAPK, PI3K, and TGF-beta, allowing CRC to continue to develop (Malki et al., 2020; Shaoping, 2013; Jiexi et al., 2021). Furthermore, researchers found that non-coding RNA can also affect the multilayered expression of genes related to CRC signal pathways through the ceRNA mechanism, sponge effect, and translation regulation, thereby influencing the occurrence and metastasis of CRC (Li et al., 2019; Wang, 2020; Huang et al., 2021; Ma et al., 2021; Gharib et al., 2024; Lee et al., 2024; Zhang et al., 2025). With the advancement of detection methods, researchers gradually realize that cancer is a complex regulatory network system involving multiple gene interactions, where a single gene often cannot explain its molecular mechanisms. With the development of omics, it helps us to simultaneously observe multiple gene interactions and impacts, thereby revealing the complex genotype-phenotype correlations in the process of cancer development, providing a new perspective for elucidating the mechanism of CRC occurrence (Mao et al., 2024).

## 2 Study of CRC on the bulk sequencing level

### 2.1 Identification of tumor biomarkers

CRC molecular biomarkers play an important role in predicting their prognosis and guiding surgery and postoperative treatment. Therefore, many studies attempt to predict the prognosis of CRC by searching for potential molecular biomarkers. For example, *NTRKK* (Westphalen et al., 2021; Martelli et al., 2022), LncRNA HIF1A-AS2 (Zhong et al., 2024), and ITGB8-AS1 (Lin et al., 2022), etc. In addition, Nakamura et al. (2022) found and verified that the combination of miR-193a-5p, miR-210, miR-513a-5p, and miR-628-3p can accurately identify patients with EOCRC through the analysis of non-coding RNA expression profile datasets. Du et al. (2023) analyzed the WGS and WES sequencing data of EOCRC and late-onset CRC (LOCRC), and found that EOCRC had a higher tumor mutation burden, and LMTK3 showed ancestral mutation differences, possibly acting as functional regulators and biomarkers driving the development of EOCRC and immune therapy responses (Du et al., 2023). Gardner et al. (2021) used NanoString immune analysis technology to analyze the expression of immune genes in EOCRC patients, and found that the expression of SAA1, C7, and CFD increased in EOCRC, with a unique, site-dependent immune microenvironment and a poor prognosis (Gardner et al., 2021). However, these research results have not been widely applied in clinical practice, with only a few biomarkers such as DNA mismatch repair (MMR) genes and CDX2 molecules being proven to be reliable prognostic markers (Dalerba et al., 2016; Popat et al., 2005).

In order to make a better judgment on the prognosis of CRC, the earliest attempt was to analyze the gene expression characteristics through DNA gene chip technology (Wang et al., 2004). Gene chip technology depicts a relatively rough situation in gene transcriptional segment expression, in which differentially expressed genes related to tumor progression are selected from the transcriptional segments as prognostic prediction markers, and similar methods can also be seen in several articles published during the same period (Eschrich et al., 2005; Salazar et al., 2011). However, these studies have not performed well either in terms of predictive efficacy or replicability, resulting in the fact that these attempts have not been widely used in the prognostic prediction of CRC.

With the development of second-generation sequencing (NGS) technology, the identification of large-scale CRC genetic variations has become possible. In 2023, Trevor A Graham's team conducted whole-genome sequencing on 2023 CRC samples, identifying more than 250 CRC driver genes, many of which had not been previously linked to CRC or other cancers (Cornish et al., 2024). (Ya-Yu Tsai, 2024) sequenced the T cell receptors (TCRs) of EOCRC and found a higher genetic diversity of TCRs in EOCRC. Additionally, genome-wide association analysis (GWAS) is also being used for the exploration of CRC susceptibility loci and molecular markers. (Broderick et al., 2007) found through GWAS that SMAD7 is involved in TGF-β and Wnt signaling pathways, and is significantly associated with the development of CRC. The following year, Malcolm G Dunlop identified rs3802842 and rs4939827 as two susceptibility loci for CRC occurrence (Tenesa et al., 2008). Park et al. (2024) found LINC02257 to be closely associated with the progression of CRC through GWAS. In the same year, Murphy et al.also identified rs186107317 and rs9991540 as two susceptibility loci for EOCRC, through GWAS (Laskar et al., 2024). Currently, more than 200 CRC susceptibility loci have been identified through GWAS analysis (Broderick et al., 2007; Tian et al., 2019; Broderick et al., 2007; Tian et al., 2019; Fernandez-Rozadilla et al., 2023; Law et al., 2024), but the related research results have not been widely used in clinical practice. The main reason is that CRC is a highly heterogeneous disease, and bulk sequencing technology relies on homogeneous cell clustering, unable to capture the heterogeneity between cells within the tumor.

### 2.2 The classification of colorectal cancer

In the process of CRC clinical diagnosis and treatment, traditional TNM or Dukes pathological staging is still the most commonly used prognostic classification system and guides clinicians in formulating diagnosis and treatment strategies. However, for patients who have undergone radical surgery, the traditional pathological staging is not accurate in predicting postoperative recurrence and metastasis. In fact, 10%–20% of stage II and 30%–40% of stage III CRC patients still experience recurrence after surgery (Alberts et al., 2005; Wang et al., 2022; Li et al., 2020a).

With the initiation of the Cancer Genome Project, human understanding of tumor genomes is deepening, and the identification of large numbers of mutations is helping with the precise classification of CRC (Wood et al., 2007). For example, The 2012 Cancer Genome Atlas Network on colorectal analyzed the gene mutations and copy number variations of 224 cases of CRC and adjacent tissues, dividing CRC into two subgroups: those with high-frequency mutations and those without. Tumors in the high-frequency mutation subgroup often originate from the right half of the colon, with frequent BRAF gene mutations, high microsatellite instability (MSI-high), and CpG island hypermethylation (CIMP-high). However, the variability in the right half of the colon is significantly less than that in the left half in terms of allelic copy number changes (Cancer Genome Atlas, 2012). Cornish et al. (2024) defined four new common subgroups of microsatellite stable (MSS) CRC through the analysis of whole-genome sequencing data from 2023 CRC samples. This type of research provided assistance in finding new molecular markers, and deepening the understanding of the physiological and pathological changes in CRC. However, this method clearly has its limitations for predicting survival prognosis (Cancer Genome Atlas, 2012; Zhao et al., 2022).

In order to overcome this limitation, De Sousa et al. (2013) utilized unsupervised clustering to analyze gene expression data of CRC from 1,100 cases from six databases, classifying CRC into three types of CCS1, CCS2, and CCS3 (CCS classification) (De Sousa et al., 2013; Guinney et al., 2015). Among these the CCS3 type is the most sensitive to chemotherapy, However, its prognosis is the worst compared to CCS2, with a high probability of recurrence after radical. This indicates that CCS3 colorectal cancer is more molecularly associated with tumor recurrence and invasiveness; In the same year, two studies on molecular subtyping of CRC based on transcriptome data were conducted. One of them analyzed the gene expression characteristics of 1,290 cases of colorectal cancer, dividing CRC into five subtypes: epithelial cell type (high expression of epithelial cell-specific genes), goblet cell type (high expression of goblet cell characteristic genes, MUC2 and TFF3), stem cell-like type (high expression of WNT pathway, low expression of differentiation-related markers), inflammation type (high expression of cytokines and interferon-related genes) and expansion-translation type (differential expression of stem cell and WNT pathway genes). Among them, the stem-like subtype had the worst prognosis (Sadanandam et al., 2013). Another study divided CRC into 6 subtypes using a similar method (Marisa et al., 2013), C1-6. Among them, C4 and C6 had the worst prognosis. Guinney et al. (2015) researchers classified CRC again based on tumor transcriptomic data, grouping them into four major Consensus Molecular Subtypes (CMS1-4) with characteristics of immune infiltration, classical WNT and MYC pathway activation, metabolic dysregulation, and stromal fibrosis, in which the fibrotic CRC subtype indicating a poorer recurrence-free survival period (Guinney et al., 2015). PD-1/PD-L1 inhibitors, EGFR targeted therapy, and targeted metabolic pathway PI3K/AKT/mTOR inhibitors can significantly improve the clinical treatment outcomes of CMS1, CMS2, and CMS3 subtypes of patients respectively. However, CMS4 subtype typically exhibits high invasiveness and metastasis, poor prognosis, shorter survival period, and high recurrence rate (Guinney et al., 2015), often found in late-onset colorectal cancer (LOCRC) (Willauer et al., 2019). Traditional chemotherapy and radiotherapy may have limited effects on this subtype. In recent years, some studies have suggested that CMS4 is the main subtype of colorectal cancer-related peritoneal metastatic (CRC-PM). The expression of structural protein Moesin is a specific determinant of CMS4 subtype, so Moesin may be a specific therapeutic target for CMS4 subtyp (Lenos et al., 2022). Therefore, more proactive and personalized treatment strategies are needed for patients with different subtypes of CRC to improve their prognosis and quality of life.

Indeed, these molecular subtypes are highly correlated, among which CCS3 type, stem-like type, C4 type, and CMS4 have the worst prognosis, and CCS3, stem-like, and C4 types are highly consistent in gene expression. Interestingly, by establishing a mouse PDX model and excluding human tumor-associated fibroblasts (TAFs), this gene expression signature also disappeared, indicating that the gene expression characteristics exhibited by these subtypes are not expressed by colon cancer cells, but rather by TAFs (Isella et al., 2015). Considering the significantly worse prognosis of these subtypes, it can be seen that the tumor microenvironment plays a crucial role in tumor metastasis and invasion.

### 2.3 Study of colorectal cancer evolution genomic

The formation of tumors as a research on the Darwinian evolution process began in the 1970s. Nowell believes that the formation of tumors is the result of a population of cells with different mutations expanding and competing with each other under the natural selection, similar to the Darwinian evolution process experienced by a species undergoing diversification and selection, known as clonal evolution (CE) (Peterc.Nowell, 1976). The CE model suggests that a normal cell, after acquiring an inducible or spontaneous genetic mutation, will undergo cell proliferation, during which it will further acquire random mutations. Through the process of natural selection, these cells will develop into a new population of mutated cells with different adaptabilities. Specifically, most of these genetic variations are harmful, so these cells will either be eliminated in resource competition or destroyed by the host’s immune system. Occasionally, a mutation confers a selective advantage to a tumor cell, which becomes a dominant subclone. The accumulation of further mutations in this subclone, along with natural selection, will drive tumor growth, leading to increased tumor heterogeneity and malignancy (Greaves and Maley, 2012). Although the mechanism of tumor evolution has made preliminary progress, the rules of genomic evolution in the process of tumor occurrence and development, as well as its relationship with clinical biological characteristics, are still not very clear. Therefore, understanding the mechanism of tumor evolution is of great significance in cancer medicine.

#### 2.3.1 Based on bulk sequencing to infer the clonal structure of CRC

Whole exome sequencing (WES) or whole genome sequencing (WGS) integrating reads depth and variant allele frequency of somatic mutations can be used to infer the tumor purity, ploidy, and local copy number of each mutation, then determining the Cancer Cell Fractions (CCF). To some extent, these data can explore the relationship between clones and subclones. For example, Banerjee et al. (2021) used WES sequencing technology to sequence left hemicolectomy, right hemicolectomy, rectal, metastatic lymph nodes, and lymph node extracolonic tumor, showing that intratumor heterogeneity (ITH) and clonal evolution play a crucial role in the occurrence and metastasis of CRC. Among them, CRC metastatic lymph nodes (LN) and lymph node extracolonic tumor (ENTD) originate from different clones. In the same year, Zhaoyang Zhong and others conducted WES and RNA-seq analysis on primary and metastatic tumors of CRC, and found that the same somatic mutations between primary tumors and metastatic tumors usually have a subclonal-clonal (S-C) evolutionary pattern, proving the existence of a common clonal origin between the two lesions (Li et al., 2021).

Additionally, In aneuploid solid tumors, whole genome sequencing (WGS) can also help to compare early and late alterations of clonal cells by determining whether SNVs occur before or after large-scale amplification processes (such as whole genome duplication, WGD) (Gerstung et al., 2020). For example, in 2021, Kim's team analyzed the whole exome sequencing (WES) data of 47 EOCRC patients and targeted deep sequencing data of 833 CRC cases, and found that *TP53* dysfunction leads to whole genome doubling (WGD) and amplification of oncogenes, thereby forming a unique genomic evolutionary path, which may also be a key factor in the onset of EOCRC (Kim et al., 2021).

#### 2.3.2 Multiple-sampling tracking the dynamic changes of clones

Due to CCF changes over time, sampling at different time points during the cloning evolution process can provide higher resolution phylogenetic relationships for subclones with lower CCF, thereby identifying individual subclones that are markedly different from other subclones. Siravegna et al. (2015) conducted intensive sampling at different time points of ctDNA in the plasma of CRC patients, using mathematical models and bioinformatics algorithms to evaluate the relationship between clonal growth kinetics in tumors and treatment. The results showed that cancer evolution is a dynamic process, with different adaptive clonal subtypes potentially reshaping the genomic information of tumors, then affecting the formulation of clinical drug use and treatment plans. Additionally, researchers characterized the spatial evolutionary patterns of clones and the formation of heterogeneity by sampling multiple regions of the same tumor or multiple tumor lesions in the same tissue (Nam et al., 2021). Li J. et al. (2020) used a multi-region sampling strategy to investigate the lineage relationships between multiple lesion tissues from the same FAP patient, as well as the genomic variations during the transformation from benign adenoma to malignant adenocarcinoma. The results indicated that somatic cell mutations in CRC begin to occur in the early stages of cancer development, even in low-grade adenomas, the number of somatic cell mutations is significantly higher than in adjacent normal tissues (LI J. et al., 2020).

The above research indicates that a large number of early mutations and driving events have already occurred before the diagnosis of colorectal cancer. If these key “culprits” can be identified in advance, it will provide an opportunity for doctors to intervene in the occurrence of colorectal cancer in advance. However, WES and WGS have many bottlenecks in low CCF conditions when sequencing the entire organization, which limits their ability to resolve the developmental relationship of clones. Therefore, it is still necessary to resolve the system development from the single-cell level in order to obtain precise understanding of clonal dynamics and tumor evolutionary history (Nam et al., 2021).

#### 2.3.3 Study of the mechanism of colorectal cancer metastasis

Although the progress in treatment of CRC continues to increase, the mortality rate caused by CRC, especially metastatic CRC, remains high. Currently, only the DNA mismatch repair, RAS (Malumbres and Barbacid, 2003), and BRAF mutation status will affect clinical decisions. The development of genomic technology provides more opportunities for analyzing the mechanisms of CRC occurrence and metastasis. Sun et al. (2019) analyzed the WES and WGS sequencing data of metastatic tissues, primary tumor tissues, and adjacent cancer tissues from CRC patients with brain metastases (BMs), indicating that BMs exhibit mutation characteristics of homologous recombination deficiency (HRD) and mismatch repair deficiency (MMRD). Further analysis shows that two DNA damage response (DDR) signals can appear in the early stages of CRC and are enhanced in the BM tissue, but eventually are eliminated in the corresponding primary CRC tissue. The specific mutations of DDR gene and the high level of microsatellite instability (MSI) in the BM strongly support the importance of DDR in the process of CRC brain metastasis. The study also identified genes related to BM (such as *SCN7A, SCN5A, SCN2A, IKZF1*, and *PDZRN4*), which carry BM-specific mutations (Sun et al., 2019). The study provides a better understanding on the gene mutations and treatment of CRC brain metastases. In the second year, Wu et al. analyzed the genomics, proteomics, and phosphoproteomics data of 480 clinical tissues from 146 Chinese CRC patients (including 70 cases of metastatic CRC, mCRC). The results showed that metastatic tumor tissues were highly similar to primary tumor tissues at the gene level, but not at the proteomic level. Kinase network analysis revealed significant heterogeneity between primary CRC and its liver metastases (Li c. et al., 2020). Hoye et al. (2022) identified five miRNAs significantly associated with metastatic progression based on NGS data from 268 samples of primary and metastatic colorectal cancer, with their expression levels negatively correlated with overall survival rates of patients. In addition, Wang et al. (2020) analyzed targeted sequencing data of EOCRC and LOCRC, showing that approximately onetenth of EO mCRC is associated with hereditary tumors, and the somatic mutation spectra between early-onset and late-onset mCRC are generally consistent. In EO mCRC, wild-type WNT pathway and mutated TGF-β pathway are associated with poor OS (Xu et al., 2020).

Due to the fact that the venous blood of the intestine mainly flows back through the portal venous system, the metastasis pattern of CRC is often considered linear, with tumor cells first entering the liver through the portal vein via microvessels, and ultimately spreading to organs outside the liver such as the lungs and brain through the systemic circulation. However, this anatomical hypothesis lacks direct genomic evidence support and fails to explain the diversity of clinical cases of metastasis, mainly due to a lack of systematic evolution and clonal reconstruction analysis for multi-organ metastasis samples matched to individuals. Regarding this issue, Xu et al. (2021) conducted whole exome sequencing on surgical specimens of multi-organ metastases from 14,000 CRC patients, detected the mutation types of lesions in multiple areas, and traced the metastatic pathways and clonal origins of each case using the methods of genomic evolution and clone reconstruction. The research results confirmed the anatomical hypothesis that CRC cells can first settle in the liver, expand clonally, and then spread to other organs such as the lungs, and revealing that in some cases tumor cells can bypass the liver and directly metastasize to other organs. Clonal reconstruction analysis showed that some metastases directly originated from late clones with additional driver mutations acquired in the primary lesion, resulting in delayed postoperative metastatic events (Chen et al., 2022).

Although there has been significant progress in the basic research of the pathogenesis of CRC, the Cancer Genome Atlas has not yet brought significant benefits to the clinical treatment of tumors, leading scientists to question the tumor genomics of bulk sequencing and think deeply on the molecular mechanisms behind tumor development and possible complex evolutionary dynamics. The possible reason is mainly that bulk sequencing technology relies on homogeneous cell clustering, which cannot capture the heterogeneity between cells within tumors and the interactions within the tumor microenvironment, and the previously recognized molecular subtypes of CRC are based on bulk transcriptome analysis, unable to identify their potential cellular diversity. The development of single-cell technology provides a new opportunity for a deeper understanding of the mechanisms of CRC occurrence.

## 3 Study of single-cell genomics in colorectal cancer

The application of single-cell technology in tumor research mainly includes three aspects, First, revealing the heterogeneity within tumors, such as helping identify and classify tumor subclonal cells, and revealing their roles in tumor progression. Second, revealing the heterogeneity of the tumor microenvironment, including the distribution, function, and interactions of different cell types within the tumor microenvironment. Third, by analyzing the transcriptome, proteome, and epigenome data of single cells to identify characteristics of different cell types such as tumor cells, immune cells, and endothelial cells, and understand their interactions in the tumor microenvironment, such as immune cell infiltration, angiogenesis, and cell-to-cell signaling.

### 3.1 CRC genetic history tracking in single-cell level

Tumor evolution is a process of continuous increase in the malignant tumor cell population. Although WGS/WES provides a higher resolution for deconstructing the evolutionary clones of tumors, the sequencing of a large number of cells in BULK-seq can result in low abundance clones being masked by high abundance clones in the sequencing results, making accurate detection impossible. Single-cell sequencing can accurately detect low abundance clones, reduce the masking effect caused by cellular heterogeneity, and consequently derive precise clonal dynamics and the evolutionary history of the tumor.

Currently, lineage tracking by optical (immune fluorescence FISH probe) or single-cell barcoding high-throughput technologies (commercial single-cell sequencing platforms led by 10X Genomics and BD) has technically achieved modeling tumor evolution *in vivo* and *in vitro*, and has been successfully used in tumor developmental studies such as lung cancer (Yang et al., 2022; Jeffrey et al., 2021), pancreatic cancer (Lee and Kang, 2021) and liver cancer (Zhou et al., 2022), as well as research related to targeted therapy of CRC tumor stem cells (CSC) (Frank et al., 2021; Cortina et al., 2017; Goto et al., 2019). However, the application of analyzing CRC developmental studies is relatively limited, and because these two technologies require the use of somatic mutation gene barcoding information, they cannot be used for lineage tracking in primary human tissue.

Single-cell genome sequencing (scWGS/scWES) can infer Copy Number Alteration (CNAs) of natural barcodes, directly applied to the retrospective lineage tracing of human tumors, thereby depicting the systematic evolutionary relationships between individual cells (Fan et al., 2021), paving the way for inferring key parameters of evolution from patient-derived somatic cells. For example, Wu et al. (2017) through conducting scWES and WGS on the cancer tissues, adenomatous polyps, and normal tissues of two CRC patients, found that colorectal adenomas and CRC are both monoclonal in origin, followed by further diversification into different subclones, (Wu et al., 2017), providing a new perspective for studying the potential genetic diversity of tumor clone evolution. Wu et al. (2022) showed through scWES sequencing studies of CRC that tumor cells originate from CRC stem cells CRCICs (CD133+), and targeted sequencing reveals specific mutations in *AHNAK2, PLIN4, HLA-B, ALK, CCDC92*, and *ALMS1*, which may be prognostic markers for CRC (Zhang et al., 2022). In addition, although numerous studies indicate that genetic mutations cause the occurrence of CRC, it is not clear whether they will lead to the metastasis and drug resistance of CRC. In 2021, the analysis of CRC scWES and bulk WES sequencing data showed a low genomic difference between primary tumors and metastatic tumors, but scWES data revealed rare mutations and defined two independent cell populations, indicating different evolutionary trajectories between primary and metastatic tumor cells (Tang et al., 2021). Recently, “Whiting and Graham (2025) used single cell RNA-seq to study the changes in the cancer cell state (cell phenotype) during the development of colorectal cancer. They found that tumor initiating mutations in the tumor may give the tumor cells a “permissive phenotype,” allowing them to transition between cell states, meaning that cancer cells may be able to metastasize without the need for specific genetic changes. At the same time, they identified the transcription factor PROX1 as being able to restrict differentiation mechanisms, so insensitivity 365 to PROX1 constraints may be an important step in acquiring a metastatic cell state (Whiting and Graham, 2025). These studies help to promptly “pinpoint” early mutations and driving events that promote tumor progression, providing an opportunity for doctors to intervene in a timely manner.

### 3.2 Single-cell level liquid biopsy of CTCs helps in early screening of CRC

The traditional diagnosis of tumors mainly relies on invasive tissue biopsy, which may result in complications such as infection and bleeding, and is also difficult to operate on certain areas such as brain or lung metastases. In addition, single-time organ biopsies often cannot fully reflect the genomic composition of all metastases in the patient’s body. In comparison, liquid biopsy (LB) as a non-invasive detection method, its biomarkers include circulating tumor DNA (ctDNA) and circulating tumor cells (CTCs), these biomarkers have shown huge potential applications in various stages of cancer (Pantel and Alix-Panabieres, 2010). Among them, CTCs mainly originate from primary or metastatic lesions of epithelial tumors, enter the bloodstream, and possess high dynamicity and high metastatic potential. Epithelial-mesenchymal transition (EMT) is an important mechanism in the progression of CRC, and CTCs are considered a powerful tool for revealing the potential of mesenchymal phenotype transformation and EMT-specific markers. Furthermore, CTCs can be repeatedly collected and monitored in real time (Carter et al., 2017). However, due to the extremely small amount of tumor markers released into the blood by small tumors (Zhou et al., 2022a), the sensitivity and specificity of the technique become key challenges.

Single-cell sequencing technology can use a small amount of circulating tumor cells (CTCs) as samples for sequencing objects, analyze the expression and mutations of tumor markers in CTCs, and compare the differences in single-cell genomics, transcriptomics, and epigenomics between CTCs, primary tumor foci, metastases, and metastatic lymph nodes, thereby reducing the interference of tumor heterogeneity and solving the sensitivity and specificity issues of the technology. In addition, this technology can also provide more specific diagnostic and prognostic markers, as well as actionable therapeutic targets (Nikanjam et al., 2022; Alix-Panabieres and Pantel, 2024; Sun et al., 2025).

Currently, the research using single-cell sequencing technology to sequence CTCs for liquid biopsy has been successfully applied to monitor the metastasis of lung cancerr (Ni et al., 2013; Carter et al., 2017), breast cancer (Cheng et al., 2019), liver cancer (Sun et al., 2021), analyze the molecular mechanisms of chemotherapy resistance, identify drug sensitivity, and elucidate immune evasion mechanisms. However, research on liquid biopsy technology for colorectal cancer CTCs combining single-cell sequencing is relatively limited (Tieng et al., 2020).

Furthermore, with the deepening of research, researchers gradually realize that besides key driver gene mutations, the accumulation of mini-drivers also affects the progression of tumors (Wu et al., 2020). The accumulation of driver gene mutations and mini-drivers may exacerbate tumor formation. For example, Campos Segura et al. (2023) showed through the analysis of CRC data in the cBioPortal database that *DOCK3, FN1, PAPPA2, DNAH11,* and *FBN2* are mini-drivers of CRC progression, and are associated with poor prognosis of CRC (Campos Segura et al., 2023).

In summary, the molecular information of CTCs, especially omics information, is undoubtedly more valuable, not only confirming that the found CTCs are indeed tumor cells, but also providing more biological information to guide clinical diagnosis and treatment, especially to help discover the differences in molecular characteristics of metastatic tumors and in situ tumors at the cellular level for better development or use of drugs. Therefore, using single-cell sequencing technology to sequence CTCs for liquid biopsy is an inevitable technological path for early diagnosis and follow-up monitoring of CRC, providing a new perspective for the early diagnosis and follow-up monitoring of CRC.

### 3.3 Study on heterogeneity of CRC in single-cell level

Heterogeneity is one of the important characteristics of malignant tumors. After multiple cell divisions during the process of tumor initiation and progression, offspring cells undergo genetic variations and changes in molecular characteristics, leading to differences in tumor cell proliferation and invasion abilities, drug sensitivity, prognosis, and other aspects (LI J. et al., 2020). As a result, inheritable diversity emerges, driving clonal evolution of cancer. However, most studies have focused on heterogeneity between different cancer patients and the emergence of single-cell sequencing technology has made it possible to understand the genetic heterogeneity at the single-cell level within cancer patients. In 2018, Roerink team’s study study showed that CRC cells have extensive genetic diversity, carrying several times more somatic mutations than normal tissue cells. Most of the mutations occur during clonal expansion in CRC cells, and the genetic information of each cell is different in CRC tissue (Roerink et al., 2018). Can Chen, (2025) conducted scRNA-seq, snMultiome-seq, and spatial transcriptome (ST) analyses on multiple-stage samples of colorectal cancer patients, systematically depicting cell heterogeneity during the progression of colorectal cancer and revealing dynamic changes in 48 cell subtypes and molecules. Additionally, the researchers comprehensively depicted a multi-stage cellular dynamic interaction network map, discovering that as the disease advances, cell interactions become more frequent and tend towards disorder, accompanied by the evolution of signal pathways involving receptor-ligand participation (Can Chen, 2025). In addition epigenetic characteristics can also affect the heterogeneity of tumors. Shuhui Bian et al. (2019) conducted single-cell multi-omics sequencing (DNA, DNA methylation, and transcriptome sequencing) on CRC patients. The results showed that the DNA methylation level in tumor cells was lower than that in adjacent normal epithelial cells, and there were differences in the methylation levels of different subclonal cell populations within the same tumor tissue, indicating that methylation heterogeneity mainly originated from DNA methylation differences between different subclonal cell populations within the same patient’s tumor (Shuhui Bian et al., 2019). Zhu et al. (2023) Jing conducted single-cell transcriptome and epigenome analysis, revealing potential heterogeneity among seemingly homogeneous cells. And during the amplification process of cell lines, the transcriptome characteristics of individual cells undergo dynamic changes. Zhenyu et al. (2024) identified two different epigenetic subtypes through single-cell chromatin accessibility (scATAC-seq) profiling, which matched completely with the known iCMS classification. Additionally, researchers have also identified key transcription factors such as *HNF4A, PPARA, FOXA3*, and *MAFK*, which play important roles in different subtypes, thus better explaining the relationship between the heterogeneity of CRC and molecular subtypes. Moreover, besides genetic factors and epigenetic characteristics, the region of colorectal cancer will also affect its heterogeneity. For example, In 2024, Wang’s team discovered that proliferative stem progenitors are significantly enriched in malignant epithelium of the left colon through single cell transcriptomics, spatial transcriptomics, and large-scale histological analysis of colon cancer. These cells co-located with Mph-PLTP cells, activated regulatory T cells (Tregs), and exhausted CD8-LAYN cells, forming a niche for metabolic reprogramming. Immune secretion (IS) process shows specific enrichment in malignant epithelium in the right colon, especially in patients with right colon cancer who smoke (Liu et al., 2024). In the same year, Cheng’s team found through single cell RNA-seq sequencing that immune and stromal cells of left and right colon cancer tissues showed that the left half of the colon displayed a stronger tumor invasive ability and worse prognosis compared to the right half. Furthermore, researchers also discovered a potential novel *MYH11+* cancer-associated fibroblast (CAF) subset that is primarily enriched in left-sided colorectal cancer. *MYH11+* CAFs may promote tumor migration through interaction with macrophages and are associated with poor prognosis in colorectal cancer (Wang et al., 2024). These research results provide important insights into understanding the single-cell level heterogeneity of the same cancer cell line, and offer new ideas and theoretical basis for developing personalized diagnosis and treatment strategies as well as tackling drug resistance in cancer therapy.

### 3.4 Study of the colorectal cancer tumor microenvironment

The tumor microenvironment, as an important part of tumors, not only contains tumor cells but also various types of cells such as immune cells, fibroblasts, endothelial cells, and adipocytes, as well as extracellular components and signaling molecules surrounding tumor cells (such as surrounding blood vessels, extracellular matrix, cytokines, and growth factors, etc.) (Wu and Dai, 2017). The cells in the tumor microenvironment may promote tumor progression through interactions with cancer cells, but little is known about the interactions between these non-cancerous cells and cancer cells. Single-cell sequencing provides a more precise cellular map for comprehensive analysis of the growth, immune infiltration, and intercellular interactions of tumor cells within cancer tissues (Qian et al., 2020). In 2020, single-cell sequencing studies on CRC found that the proportion and functional state of T cells and B cells in CRC are changed compared to normal tissues. In early CRC, B cells were identified as expressing tumor suppressor factors called pre-B cells, while in late-stage CRC, B cells tended to develop into plasma cells. It is believed that the different roles of B cells in tumors may be due to the diverse roles played by different subtypes of B cells in tumors (Wang et al., 2021). In the same year, Tang team performed single-cell multi-omics sequencing on samples from 21 microsatellite stable CRC patients and six normal individuals. The results showed that somatic copy number alterations (SCNA) were commonly present in immune cells, fibroblasts, and endothelial cells in both tumor and normal tissues, with a significantly higher proportion of SCNA in fibroblasts in tumors compared to adjacent normal tissues. This study demonstrates the presence of widespread genomic alterations in the stromal cells of the tumor microenvironment of CRC (Zhou et al., 2020). Chen et al. (2021) discovered through scRNA-seq, WGS, and the study of immunologic pathology, that the mechanisms of colorectal adenoma-like and serrated polyps have significant differences: the former originates from mutations caused by constantly updating stem cell DNA replication, while the latter originates from cellular biochemical processes caused by colonic surface stimulation from within the lumen. Among them, the serrated adenoma is characterized by an increased number of CD8^+^ T cells in the immune microenvironment, and this change in the immune microenvironment occurs earlier than the occurrence of genomic hypermutation (Chen et al., 2021). Guo et al. (2023) conducted a comprehensive analysis by immunoscore, multiplex immunohistochemistry, whole exome sequencing, transcriptome, and single-cell sequencing of 869 Chinese CRC patients, systematically assessing the heterogeneity of the tumor immune microenvironment under different genomic backgrounds, providing a theoretical basis for CRC prognosis prediction, drug response, and genomics-based personalized targeted and immunotherapy. In the same year, Deng et al. revealed through the analysis of single-cell transcriptome data that the potential mechanism of PD-1 resistance in dMMR/MSI-high colorectal cancer is the IL-1B driven inflammatory microenvironment and residual Tregs, providing new immunotherapy targets for dMMR/MSI-high colorectal cancer. The personalized treatment developed based on this mechanism significantly improved the objective response rate of PD-1 inhibitors therapy. This treatment method has been included in the 2024 version of NCCN treatment guideliness (Li et al., 2023a). By the end of 2024, Zhang’s team divided colorectal cancer patients into six groups based on the cellular composition of the tumor microenvironment, depicting the different tumor microenvironment characteristics of each group of patients and their corresponding immune escape mechanisms, providing a theoretical basis for personalized immunotherapy for colorectal cancerr (Chu et al., 2024).

Tumor immunotherapy targets specific immune-related markers for immune intervention, but the markers that can be used in clinical practice are difficult to determine. Despite high-throughput sequencing being able to identify a large number of biomarkers, due to the complexity of the immune system, the number of truly usable clinical markers is very limited. Zhang et al. (2020) used single-cell transcriptome sequencing technology to analyze the immune cell subsets in tumor tissues of CRC patients, which identified the TAMs subset and DCs subset that play a key role in the interaction between cells in the tumor microenvironment, and identified the myeloid cell subset similar to human myeloid cells from a mouse tumor model, and verified that anti-CSF1R therapy tends to eliminate pro-inflammatory macrophages while retaining pro-tumor angiogenesis macrophages, and anti-CD40 therapy tends to activate the cDC1 subset, thereby activating Bhlhe40+ Th1-like cells and CD8^+^ memory T cells, clarifying the mechanism of current clinical myeloid-targeted immunotherapy (Zhang et al., 2020). Qi et al. (2022) integrated publicly available scRNA-seq and bulk RNA-seq datasets, spatial transcriptome data, FACS, and transcriptome data of immunotherapy, revealing the interaction between FAP + fibroblasts and SPP1+ macrophages in the formation of a pro-fibrotic tumor microenvironment, which can serve as a potential target for CRC treatment.

How immune cells coordinate CRC metastasis in space, and whether the microenvironment of metastatic tumor cells is different from that of primary tumor cells, remains largely unknown. Single-cell sequencing technology can identify unique gene mutations and transcriptomic features of invasive metastatic tumor cells in CRC patients, identify key factors in tumor metastasis, and identify molecular mechanisms in the process of tumor metastasis. Wu et al., (2022) integrated single-cell transcriptomics, spatial transcriptomics, and other technologies, depicting the spatiotemporal map of single cells in CRC liver metastasis, revealed the molecular pathways and metabolic characteristics of the specialized subpopulation of macrophages “domesticated” in metastatic lesions, providing new clues for understanding the tumor metastasis microenvironment. Li et al. (2023b) identified a TCF21high pericyte subpopulation in tumor-surrounding cells of colorectal cancer liver metastasis patients. This subpopulation promotes tumor cell migration to the liver by reshaping the extracellular matrix stiffness of the blood vessel's surrounding cells. The study further discovered that inhibiting integrin α5 (ITGA5) can disrupt the metastatic microenvironment by regulating DNMT1 methyltransferase activity, blocking TCF21 expression. This finding provides theoretical support for developing anti-metastasis therapies targeting the vascular microenvironment (such as ITGA5 inhibitors), with related drugs currently in clinical trial phase (Li et al., 2023b). Liang et al. (2024) discovered that in CRC patients with liver metastasis, the proportion of M2 macrophages increases. Highly metastatic CRC cells release exosomes rich in miR-106a-5p, which promote M2 macrophage polarization by inhibiting SOCS6 and activating the JAK2/STAT3 pathway. These M2 macrophages in turn promote liver metastasis of CRC. Clinically, elevated levels of miR-106a-5p in plasma exosomes are associated with liver metastasis and poor prognosis. Li et al. (2025) analyzed the transcriptome characteristics of colorectal cancer and its peritoneal metastasis, revealing how the reshaping of tumor cells and TME promotes peritoneal metastasis, providing important insights into the mechanisms of peritoneal metastasis in CRC, and possibly offering new strategies for preventing and treating peritoneal metastasis in clinical practice (Li et al., 2025).

### 3.5 The correlation research of single-cell multi-omics reveals the evolutionary mechanisms of CRC

In addition to genetic factors, factors such as cell state, epigenetic characteristics, spatial distribution, and the microenvironment of cell growth can all affect the evolution of tumors (Nam et al., 2021). Therefore, single-cell multi-omics association studies are of great significance for dissecting the evolution mechanism of CRC. For example, CRC follows a stereotype progression from normal to atypical and then to cancer, making it an ideal system for studying malignant transformation of tumors. Research on the evolution mechanism of CRC helps to elucidate the genetic regulatory mechanisms during its malignant transformation process, identify patients in the precancerous lesion stage, then implement effective precancerous interventions and propose new diagnosis and treatment strategies. Li J. et al. (2020) integrated scRNA-seq and bulk sequencing technologies, constructing the transcriptome dynamic changes during adenoma development and adenoma-to-adenocarcinoma transition, revealing that adjacent lesions in FAP patients may originate from the same cell, and metabolic reprogramming occurs in precancerous adenomas, implying that precancerous lesions already have metabolic features of cancer (Li et al., 2020b). Becker et al. (2022) analyzed polyps, normal tissues, and CRC samples using scATAC-seq and snRNA-seq. The results indicate that the entire process of CRC development from normal tissue to adenoma and then to cancer is accompanied by orderly opening and closing of chromatin accessibility (Becker et al., 2022). A map depicting the changes in cell composition and cell states during the evolution of a healthy colon into adenoma and then to CRC was created.

## 4 Challenges and future direction

The bulk sequencing technology, with advantages such as low cost, high throughput, fast speed, and large amount of information, is widely used in the study of the occurrence and development mechanisms of CRC. With the continuous optimization of sequencing technology and further reduction of costs, personalized medicine will become an important trend in CRC treatment.

However, due to bulk sequencing involving the sequencing of mixed samples of a large number of cells, it is unable to accurately capture the specific mutations and expression changes of individual cells or subclones. Moreover, CRC exhibts high intratumoral heterogeneity and subclonal mixing, making it difficult to decipher the heterogeneity within CRC using bulk sequencing techniques. The single-cell sequencing technology has made important progress in deciphering the mechanisms of colorectal cancer development and progression, due to its many advantages in analyzing cell heterogeneity, cell development, discovering new cell types, studying transcription dynamics, and rare cells (Navin et al., 2011; Hou et al., 2012; Zhang et al., 2020; Yin et al., 2025). However, single-cell sequencing technology also has its limitations.

Firstly, it is batch effect, such as different library construction platforms, reagent batches, and sequencing times introducing systematic bias, leading to incomparable data and masking the true biological signals. To address this issue, batch allocation and algorithm correction can be balanced, and a uniform quality control index and preprocessing steps can be established to reduce the batch effects of the data. Then, it is the inability of single-cell sequencing to provide spatial information of cells in tissues, limiting research on the interaction between tumor cells and the immune microenvironment. The combination of spatial transcriptomics and single-cell sequencing technologies will effectively locate the spatial distribution of gene expression (Li et al., 2022; Qi et al., 2022). Therefore, the combination of spatial transcriptomics, epigenomics, proteomics, single-cell transcriptomics, and other multi-omics technologies will effectively reconstruct the dynamic interaction network of cells in tissues (Shuhui Bian, 2019; Li et al., 2020b; Becker et al., 2022). In addition, in the study of tumor evolution and metastasis, obtaining continuous tumor samples is very difficult, so the combination of single-cell sequencing technology and CRISPR-based lineage tracing provides a breakthrough tool for high-resolution tracking of the clonal evolution mechanism of cancer cells, understanding tumor heterogeneity, metastasis pathways, and screening of therapeutic target points (Yang et al., 2022). Next, is the complexity of data analysis. Data preprocessing involves normalizing gene expression matrices, dimensionality reduction, and clustering, parameter selection, and over-reliance on marker genes for clustering, all of which may result in bias. Currently, with the development of new algorithms, the impact of technical noise on gene expression levels will be effectively reduced, improving clustering stability and simplifying analysis steps (He et al., 2022). Finally, the cost of single-cell sequencing is high, so developing multi-threaded single-cell sequencing technology to batch process hundreds of samples will significantly reduce the cost of single-cell sequencing (Perez et al., 2022).

In the future, it is believed that with the continuous improvement of omics technology, it will help to achieve a more in-depth and accurate analysis of the molecular mechanisms of colorectal cancer progression, providing new ideas for the diagnosis and treatment of CRC. At the same time, the cost of sequencing technology will gradually decrease, making it more affordable for more laboratories, thereby promoting the application of this technology in CRC basic research and clinical practice.
